# Seronegative Neuromyelitis Optica Spectrum Disorder in a Six-Year-Old Patient: A Case Report

**DOI:** 10.7759/cureus.106714

**Published:** 2026-04-09

**Authors:** José Luis Pinacho-Velázquez, Jorge Jesús Rueda-Velázquez, Oscar Martínez-Juárez, Erika Perez Sanjulian-Krasovsky, Fernanda Esmeralda Pérez-Trueba

**Affiliations:** 1 Pediatrics, Hospital Angeles Lindavista, Mexico City, MEX; 2 Pediatric Neurology, Hospital Angeles Lindavista, Mexico City, MEX; 3 Radiology, Hospital Angeles Lindavista, Mexico City, MEX; 4 Education, Hospital Angeles Lindavista, Mexico City, MEX

**Keywords:** aqp4, igg, neuromyelitis optica spectrum disorder, pediatric, seronegative

## Abstract

Neuromyelitis optica spectrum disorder (NMOSD) is a rare demyelinating disease of the central nervous system (CNS) in children. Its main clinical manifestations are optic neuritis and myelitis. In most cases, it is associated with suggestive magnetic resonance imaging (MRI) and positive serology (AQP4-IgG).

We report a six-year-old girl with acute asymmetric quadriparesis, bilateral decreased visual acuity, and sudden loss of sphincter control. Although AQP4-IgG testing was negative, MRI demonstrated findings consistent with NMOSD. Consequently, she received high-dose methylprednisolone, intravenous immunoglobulin, and early rehabilitation, resulting in substantial neurological recovery.

High clinical suspicion for NMOSD in children, particularly in the presence of bilateral optic neuritis and longitudinally extensive transverse myelitis (LETM), should prompt early treatment, even in seronegative cases, to prevent permanent neurological deficits.

## Introduction

Neuromyelitis optica spectrum disorder (NMOSD) is an immune-mediated astrocytopathy characterized by severe inflammatory attacks predominantly affecting the optic nerves and spinal cord [1,2]. NMOSD occurs in less than 1% of the white population and accounts for 20-48% of demyelinating diseases in non-white populations, with a predominance in women (>80%) and an average age of onset of 30 years [3]. The disease is driven by autoantibodies against aquaporin 4 (AQP4 IgG), which initiate complement-mediated astrocytic injury, disruption of the blood-brain barrier, and secondary demyelination [4]. This pathophysiological mechanism distinguishes NMOSD from multiple sclerosis (MS), in which the primary target is the oligodendrocyte, and from MOG antibody-associated disease (MOGAD), where the immune response is directed against myelin oligodendrocyte glycoprotein and typically results in a different clinical course, imaging pattern, and prognosis [5].

The 2015 International Panel for NMO Diagnosis (IPND) established criteria that emphasize the central role of AQP4 IgG seropositivity in confirming NMOSD. In seronegative individuals, diagnosis requires the presence of at least two core clinical features, characteristic magnetic resonance imaging (MRI) findings, evidence of dissemination in space, and exclusion of alternative demyelinating disorders [6]. While these criteria are well defined, their application in children is particularly challenging.

Pediatric-onset NMOSD is rare and often presents atypically. Current data suggest that it accounts for 3-5% of all NMOSD cases, with higher rates of seronegativity and substantial clinical overlap with MS, MOGAD, acute disseminated encephalomyelitis, and other inflammatory myelopathies [7]. These factors complicate early recognition and may delay the initiation of appropriate immunotherapy. Moreover, the radiological features of NMOSD in children can be less specific, and the clinical course may differ from that observed in adults, further increasing diagnostic uncertainty [8].

Given these challenges, a detailed characterization of seronegative pediatric cases is essential to refine diagnostic approaches and improve clinical decision‑making. Reports of such cases contribute to the growing understanding of how NMOSD manifests in early childhood, highlight the limitations of current biomarkers, and underscore the importance of integrating clinical, radiological, and immunological data [1].

In this context, we present the case of a six‑year‑old child with a clinical syndrome and MRI findings highly suggestive of NMOSD despite negative serological testing. This case highlights the diagnostic complexity of seronegative pediatric presentations and underscores the importance of early treatment initiation, even in the absence of confirmatory antibody assays.

## Case presentation

A six-year-old girl presented to the emergency room after waking up with asymmetrical generalized muscle weakness that began in the arms and developed in three hours. It covered all four limbs. On admission, she had autonomic breathing, bradycardia (HR 64x’), and systolic hypotension (SP 68 mmHg). She was awake, alert, oriented, and she recounted her symptoms in fluent language. Symmetrical pupils of 3 mm with accommodation reflex were present, but there was an absence of bilateral direct and consensual pupillary response. Vision was blurred; with his right eye, she could only count the explorer's fingers at 2 meters, and with his left eye, at 1 meter. She had pain on ocular mobilization, and the optic disc was pale and without edema or bleeding. Color vision was not evaluated. There was no involvement of other cranial nerves. Right brachial muscle strength was 2/5 and left 1/5; right leg 3/5 and left leg 0/5. She was hypotonic in all four limbs and developed hypotrophy with left distal predominance. Stretch reflexes were absent (0/4) in all four limbs. She had no anal reflex, nor abdominal cutaneous or Babinski. A pain sensitivity level was found at C8-T1, and a flaccid neurogenic bladder was present. A month earlier, she had a burning papulovesicular rash in the left L5 dermatome.

Visual evoked potentials to patterns showed prolonged latencies in both eyes, with P100 at 136 msec, maintaining a normal amplitude. Auditory potentials were normal.

AQP-4 (cell binding assay) antibodies were negative, and no MOG antibodies were measured. Lupus anticoagulant was present in 1:100 and 1:1000 dilutions of 1.10 U and 1.20, respectively. Beta-2 microglobulin 995 ug/L was within normal ranges. Anticardiolipins with ELISA indicated that IgM 2 MPL/U/ml acute phase was negative; IgG 16 UGPL exceeded the upper limit of the reference values in relation to the demyelinating process. A serum PCR was positive for varicella-zoster virus (VZV) infection. Cerebrospinal fluid (CSF) was not obtained.

**Figure 1:**
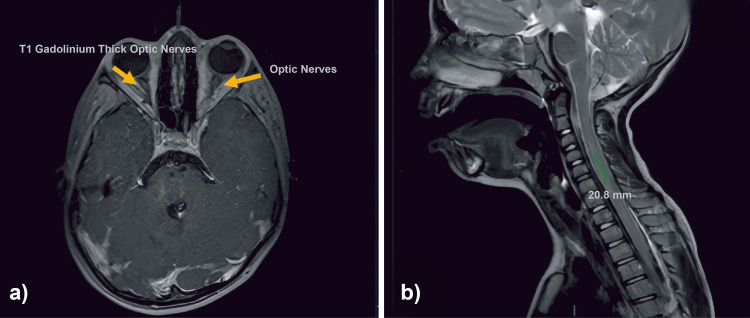
Representative images from the patient's magnetic resonance imaging (MRI). a) Axial and coronal MRI of the orbits. On the axial T1‑weighted post‑contrast sequence, bilateral intraorbital optic nerve enlargement with smooth, longitudinal enhancement is observed, confined to the intraorbital segments on this slice, with no visible involvement of the canalicular, intracranial, or chiasmal portions. On the coronal T2‑weighted sequence, diffuse thickening of both optic nerves (>5 mm) is evident (arrows). In the contrast‑enhanced phase, bilateral optic nerve enhancement is present, and a target sign is noted. Additional supratentorial lesions are visible, with probable thalamic involvement, supporting multifocal CNS demyelination. b) Sagittal cervical spine MRI. The T2‑weighted sequence demonstrates a 20.8 mm centrally located hyperintense lesion, corresponding to approximately 1.5 vertebral segments, involving the anterior and central portions of the spinal cord. Additional longitudinal hyperintensities are visible from C3 to C5, spanning more than two vertebral bodies. These lesions enhance following contrast administration and represent areas of myelin involvement. No associated cord expansion is observed.

Based on the clinical picture, magnetic resonance findings (Figure 1) and differential diagnosis (Table 1), the diagnosis was considered compatible with seronegative NMOSD and spinal shock, so five doses of methylprednisolone of 30 mg/kg/day were initiated, as well as intravenous human immunoglobulin of 400 mg/kg/day for five days without adverse effects, in addition to motor rehabilitation on Day 3. Methylprednisolone was replaced with oral prednisone 1 mg/kg/day with a tapering regimen until withdrawal eight weeks later.

**Table 1:** Differential Diagnosis. MOGAD: Myelin oligodendrocyte glycoprotein antibody-associated disease; MS: multiple sclerosis; ADEM: acute disseminated encephalomyelitis; VZV: varicella-zoster virus; LETM: longitudinally extensive transverse myelitis; NMOSD: neuromyelitis optica spectrum disorder

Condition	Supporting Features	Excluding Features
MOGAD	Bilateral optic neuritis, longitudinal myelitis	MRI pattern not typical.
MS	Optic neuritis possible	No periventricular Dawson’s fingers; LETM
ADEM	Pediatric age, encephalopathy	No encephalopathy; focal LETM
VZV myelitis	Recent rash, positive PCR	Optic neuritis + LETM pattern more consistent with NMOSD
Transverse myelitis (idiopathic)	LETM possible	Bilateral optic neuritis strongly favors NMOSD

The clinical course was favorable: breathing function was never compromised, and vital signs normalized. Around 48 hours after starting treatment, she began to regain muscle strength, stand and walk with support, regain sphincter control, and have the cervical sensory level disappear, in addition to improving vision. She recovered 3/5-4/5 proximal and intermediate brachial strength, but distal strength decreased to 0/5 bilaterally; crural strength was 5/5 on the right and 4/5 on the left. She developed hyperreflexia with clonus, a left Babinski sign, and recovery of the anal reflex. The anesthetic level disappeared, and vibratory and positional sensitivity returned. She also recovered pupillary reflexes. Four weeks later, visual acuity was 20/25 in both eyes (Snellen table), normal reflexes to stretch in the arms (2/4), and exaggerated reflexes in the legs (4/4), with left predominance and Babinski. Metries, diadochokinesia, and gait with support were developed. She was clinically surveilled for six years and currently maintains pyramidal signs with weakness 4/5 in the left hand, hyperreflexia ¾, and normal gait. Her Expanded Disability Status Scale (EDSS) score was ambulatory score 0 and functional system score 1 (pyramidal release without disability). Bathyesthesia and stereognosia are present, and the Romberg sign is absent; she has left arm allodynia. Visual acuity is 20/20 in both eyes (Snellen), and the optic disc is yellow. She has not had relapses and practices sports (swimming, flag football, horseback riding, basketball) and dance (jazz and salsa).

For clarity in the clinical context, a day-by-day timeline is provided (Table 2), along with a summary of key clinical findings and treatment responses (Table 3).

**Table 2:** Timeline of Symptom Evolution. VZV: varicella-zoster virus

Time Point	Event/Symptom
Day -30	L5 dermatome vesicular rash (VZV)
Day 0 (morning)	Onset of arm weakness → progression to quadriparesis within 3 hours
Day 0 (ER arrival)	Bradycardia, hypotension, visual loss, areflexia, neurogenic bladder
Day 1	MRI obtained; treatment initiated
Day 2	First signs of motor recovery
Day 3	Rehabilitation started
Week 4	Visual acuity recovery, reflex normalization
Year 6	Long-term outcome

**Table 3:** Summary of Core Clinical Features, Diagnostic Evidence, and Treatment Response. NMOSD: neuromyelitis optica spectrum disorder; LETM: longitudinally extensive transverse myelitis; MS: multiple sclerosis; TM: transverse myelitis; MOGAD: Myelin oligodendrocyte glycoprotein antibody-associated disease; ADEM: acute disseminated encephalomyelitis; VZV: varicella-zoster virus

Domain	Key Findings	Clinical Interpretation
Initial neurological presentation	Acute severe deficits	Classic severe NMOSD attack pattern involving optic nerves and cervical cord.
Optic nerve involvement	Bilateral optic neuritis	Strongly supports NMOSD over MS or idiopathic TM.
Spinal cord involvement	LETM	LETM is a core NMOSD feature; pattern is less typical for MOGAD.
Serological findings	AQP4-IgG negative; MOG-IgG not validated.	Seronegativity increases diagnostic uncertainty; pediatric cases are more often seronegative.
Differential diagnosis considerations	MS, MOGAD, ADEM, VZV myelitis	NMOSD remained the most consistent diagnosis despite incomplete exclusion of alternatives.
Acute treatment	High-dose IV methylprednisolone, IVIG, and early rehabilitation	Early aggressive therapy likely contributed to rapid neurological improvement.
Short-term response	Rapid motor and visual recovery	Response compatible with NMOSD attack treatment; cannot confirm causality.
Long-term outcome (6 years)	Excellent functional recovery; no relapses	Monophasic course; supports a favorable prognosis despite initial severity.

## Discussion

NMOSD in childhood remains a diagnostic challenge due to its low prevalence, heterogeneous presentations, and substantial overlap with other inflammatory demyelinating disorders. Pediatric cases account for only 3-5% of all NMOSD diagnoses, and the disease is more frequently observed in females and non-Caucasian populations, features consistent with our patient’s demographic profile [1]. The rarity of pediatric NMOSD often delays recognition, particularly when the initial presentation includes symptoms common to MS, MOGAD, acute disseminated encephalomyelitis (ADEM), or infectious myelitis [1,3].

Our case illustrates this challenge. She presented with a classic and severe case of NMOSD, characterized by bilateral optic neuritis and longitudinally extensive transverse myelitis (LETM) spanning three cervical segments. These clinical findings were accompanied by severe neurological deficits, asymmetric quadriparesis, decreased visual acuity, and loss of sphincter control, consistent with the severe attack pattern typically observed in NMOSD. MRI demonstrated bilateral optic nerve thickening with a target sign enhancement pattern and central/anterior cervical cord involvement, both of which align with imaging features described in seropositive and seronegative NMOSD [2,9].

A major diagnostic challenge in this case was AQP4 IgG seronegativity. Although AQP4 IgG is a key biomarker in NMOSD, seronegativity is more common in children, with up to one-third of pediatric patients initially testing negative [1,6]. The 2015 IPND criteria permit diagnosis in seronegative individuals when two or more core clinical features are present, alternative causes are ruled out, and MRI findings are characteristic of NMOSD [6]. In this case, the exclusion of alternative causes was not achieved. For this patient, seronegative NMOSD is considered a clinical diagnosis supported by imaging and partial exclusion of mimics, rather than a failure of antibody testing. The absence of AQP4-IgG does not rule out the diagnosis but emphasizes the heterogeneity of NMOSD and the need for comprehensive evaluation, especially in pediatric populations, where MOGAD overlap is common.

Two important diagnostic limitations must be acknowledged. First, CSF analysis was not performed, which restricts the ability to differentiate NMOSD from MS, ADEM, and infectious myelitis. CSF pleocytosis, elevated protein, or the absence of oligoclonal bands would have strengthened diagnostic confidence. The lack of CSF data, therefore, represents a meaningful limitation in the diagnostic workup.

Second, MOG IgG testing was not performed using a live cell-based assay, the current gold standard. Given the patient’s age and bilateral optic neuritis, MOGAD remains an important consideration in the differential diagnosis. However, several features favor NMOSD over MOGAD: the axial spinal cord pattern (central/anterior rather than H sign), the optic nerve enhancement pattern, and the monophasic six-year course without relapses. Even so, the absence of validated MOG IgG testing limits the ability to definitively exclude MOGAD. Despite these limitations, the overall clinical, radiological, and temporal profile strongly supports a diagnosis of seronegative NMOSD.

During acute episodes of NMOSD, intravenous corticosteroids are recommended, with proton pump inhibitors and thromboprophylaxis. In severe cases, plasma exchange may be used, or intravenous immunoglobulin may be considered in conjunction with corticosteroids. To prevent episodes, chronic immunosuppression with rituximab or its anti-CD20 alternatives is recommended if necessary. Azathioprine and mycophenolate are also useful alternatives; however, their safety profiles limit their use to regions where monoclonal antibodies are unavailable [9]. The patient’s rapid and substantial response to high-dose corticosteroids and intravenous immunoglobulin, with early motor recovery and near-complete restoration of visual function, is consistent with the expected response to immunotherapy in NMOSD [10]. Long-term follow-up demonstrated excellent functional outcomes, with an EDSS score of 1 and full visual recovery, underscoring the importance of early recognition and prompt treatment initiation.

This case highlights the need for heightened clinical vigilance when evaluating pediatric patients with bilateral optic neuritis and LETM, even in the absence of confirmatory serological markers. It also emphasizes the importance of comprehensive diagnostic evaluation, including CSF analysis and validated MOG IgG testing, to improve diagnostic accuracy. Early, aggressive immunotherapy remains essential to prevent irreversible neurological injury and optimize long-term outcomes in pediatric NMOSD.

## Conclusions

This case illustrates a demyelinating syndrome consistent with NMOSD but complicated by AQP4 seronegativity, emphasizing the diagnostic uncertainty often seen in pediatric cases. Although the clinical and radiological features strongly indicated NMOSD, the absence of AQP4 antibodies, the lack of cerebrospinal fluid analysis, and the unavailability of validated MOG-IgG testing are significant limitations in the diagnostic process. The patient experienced considerable neurological improvement after receiving acute immunotherapy, but this should be interpreted cautiously; it suggests an association in this case rather than definitive evidence of treatment effectiveness. Ultimately, this report highlights the importance of comprehensive evaluation in children with demyelinating syndromes, including CSF studies and standardized MOG antibody testing, to differentiate between overlapping conditions such as NMOSD, MOGAD, MS, and post-infectious myelitis. Rather than serving as a definitive indicator of seronegative NMOSD, this case underscores the complexity and nuance of diagnosing demyelinating diseases in early childhood.
